# Transcription dynamics of heat-shock proteins (Hsps) and endosymbiont titres in response to thermal stress in whitefly, *Bemisia tabaci* (Asia-I)

**DOI:** 10.3389/fphys.2022.1097459

**Published:** 2023-01-13

**Authors:** Mritunjoy Barman, Snigdha Samanta, Bulbul Ahmed, Soumik Dey, Swati Chakraborty, M.G. Deeksha, Subham Dutta, Arunava Samanta, Jayanta Tarafdar, Deepayan Roy

**Affiliations:** ^1^ Department of Agricultural Entomology, B.C.K.V, Mohanpur, West Bengal, India; ^2^ GD Goenka University, Gurgaon, Haryana, India; ^3^ Faculty Centre for Agriculture Rural and Tribal Development (ARTD), RKMVERI, Ranchi, India; ^4^ Department of Plant Pathology, B.C.K.V, Nadia, West Bengal, India; ^5^ Division of Entomology, I.C.A.R-Indian Agricultural Research Institute, New Delhi, India

**Keywords:** whitefly, endosymbionts, qRT-PCR, heat-shock protein, stress

## Abstract

The sweet potato whitefly, *Bemisia tabaci* (Gennadius), is one of the several species complexes of whitefly that are currently significant agricultural pests. *Bemisia tabaci* infests more than 600 plant species and thrives under a wide range of temperature conditions. In addition to the direct damage caused by sucking plant sap, it vectors several plant viruses. Heat-shock proteins play a pivotal role in enabling the insect to extend its geographical location, survival, and reproduction under different stress conditions. *B. tabaci* harbours several endosymbionts under the genera *Portiera*, *Rickettsia*, *Hamiltonella*, *Wolbachia*, *Arsenophonus*, *Cardinium*, and *Fritschea* that directly or indirectly affect its fitness. By accelerating cuticle biosynthesis and sclerotisation, symbiotic microbes can reduce or enhance tolerance to extreme temperatures and detoxify heavy metals. Thus, symbionts or microbial communities can expand or constrain the abiotic niche space of their host and affect its ability to adapt to changing conditions. The present study delineates the effect of thermal stress on the expression of heat-shock genes and endosymbionts in *B. tabaci*. Studies of the expression level of heat-shock proteins with the help of quantitative real-time polymerase chain reaction (qRT-PCR) showed that heat- and cold-shock treatment fuels the increased expression of heat-shock proteins (Hsp40 and Hsp70). However, Hsp90 was not induced by a heat- and cold-shock treatment. A significant decrease in the relative titre of secondary endosymbionts, such as *Rickettsia*, *Arsenophonus*, and *Wolbachia*, were recorded in *B. tabaci* upon heat treatment. However, the titre of the primary symbiont, *C*. *Portiera*, was relatively unaffected by both cold and heat treatments. These results are indicative of the fact that Hsp genes and endosymbionts in *B. tabaci* are modulated in response to thermal stress, and this might be responsible for the adaptation of whitefly under changing climatic scenario.

## 1 Introduction

The whitefly, *Bemisia tabaci*, is an economically important agricultural pest causing huge damage to crops worldwide. They inflict damage to plants directly and as a vector of several hundred viruses, most of which belong to the genus *Begomovirus* (>320 species), and other economically important viruses belonging to the genera *Ipomovirus*, *Carlavirus*, *Crinivirus*, *Torradovirus*, and *Polerovirus* (Bedford et al., 1994; Jones, 2003; Mugerwa et al., 2021; Navas-Castillo et al., 2011). There are many species and/or biotypes of whiteflies, each with its own preferences for host plants, virus-transmitting abilities, and insecticide resistance (Barman et al., 2022a; De Barro et al., 2011; Gilbertson et al., 2015; Perring, 2001). A wide host adaptability and virus transmission ability make it one of the 100 most dreadful alien invasive species (Barman et al., 2022b; Hogenhout et al., 2008; Lowe et al., 2000). This polyphagous pest has adapted easily to varied temperature regimes across the world, such as in India, ranging from chilling cold temperatures in the hills to oppressively high temperatures in the deserts (Barro et al., 2011; Singh et al., 2012).

Temperature is one of the important determinants of abundance and geographical distribution of every ectotherm including insects (Huey and Kingsolver, 1993). When it comes to the invasive trait of *B. tabaci*, its heat-resistance ability is considered to be one of the underlying reasons (Cui et al., 2008; Lü and Wan 2008; Wan et al., 2009). Insects in general respond to elevated temperatures and other stresses with increase in synthesis of heat-shock proteins (Hsps) (Zhao and Jones, 2012). The role of Hsps in heat/cold stress adaptation, metamorphosis, and developmental responses in other insects is well documented (Waters and Rioflorido, 2007; Aevermann and Walters, 2008; Waters et al., 2008; Pan et al., 2017; Qin et al., 2018; Xiong et al., 2018; Yu et al., 2018; Parsell and Lindquist, 1993). Based on their molecular weight and homologous relationship, the Hsps are divided into five families and they are Hsp100, Hsp90, Hsp70, Hsp60, and small heat-shock proteins (sHsps) (Li et al., 2009). Stress proteins, such as the Hsps, are a potential candidate responsible for the wide adaptability of whitefly across different geographical niches (Wolfe et al., 1999; Salvucci et al., 2000; Lin et al., 2007; Cui et al., 2008; Lü and Wan, 2008; Mahadev et al., 2009). The mechanism by which the Hsps acts as molecular chaperones is quite distinctive; they stabilize proteins to enable host survival under heat-stress conditions by safeguarding the integrity of the host cell and their homeostasis (Jakob et al., 1993).

Considering the change in the climatic scenario of the Indian sub-continent due to imperious human activities, it is rational to detect the differentially expressed genes under thermal stress condition for better understanding of the tolerance capacity of *B. tabaci* to varying temperature conditions, thus delineating the underlying mechanism for niche expansion of this pest across India. In addition, symbiotic bacteria also have an important role to play in improving the fitness of their host, enabling it to sustain under novel climatic conditions (Wernegreen and Moran 2001)**.** In accordance with other plant-sucking insects, *B. tabaci* harbours a diversity of symbionts, which enrich hosts’ nutrient-poor diet by synthesizing essential amino acids (Douglas 1989; Baumann 2005; Barman et al., 2020). Endosymbionts are generally classified into two major classes: primary symbionts/P-symbionts and secondary symbionts/S-symbionts (Baumann 2005; Feldhaar 2011). *Candidatus Portiera aleyrodidarum* (hereafter, *Portiera*), being a primary symbiont, occurs in all individuals. Secondary symbionts like *Hamiltonella, Arsenophonus*, *Cardinium*, *Wolbachia*, and *Fritschea* participate in functions that may not be necessary for the survival of the host but renders noticeable influence on its biological adaptation and ecological requirements (Ali et al., 2018; Feldhaar 2011; Khan et al., 2020). *Portiera* is described to be involved in the synthesis of nutrients such as essential amino acids (EAAs) and carotenoids, which are not present in a phloem diet (Cheng et al., 2016; Ferrari and Vavre, 2011; Su et al., 2014). Symbionts like *Hamiltonella* and *Arsenophonus* have been reported to be associated with the transmission of plant viruses (Gottlieb et al., 2010; Rana et al., 2012). *Rickettsia* is also reported to induce genes required for thermo tolerance in whitefly (Brumin et al., 2011). Reference has also been drawn indicating the obligate symbionts as “Achilles’ heel” from the perspective of temperature change (Corbin et al., 2017). Variations in the symbiont titre also have a significant impact on the insect fitness (Ali et al., 2019). For example, in *Aphis craccivora*, the quantity of *Buchnera* decreases under both low- and high-temperature conditions, which in turn negatively influences aphid reproduction (Chen et al., 2009).

The effect of heat stress in *B. tabaci* has been explored in the viewpoint of survival and reproduction (Byrne and Bellows 1991; Cui et al., 2008; Wolfe et al., 1999). Keeping these points in mind, the present research experiment was envisioned to assess the survivability of whitefly under sub-optimal and supra-optimal temperature conditions. In particular, we discussed certain key research priorities to shed light on the complex interaction between insect functioning, their microbial communities, and the Hsps gene. Primarily, the following questions were addressed: 1) what are the changes in the expression pattern of three Hsps (Hsp40, Hsp70, and Hsp90) under temperature stress conditions? 2) What are the relative changes in the symbiont titre harboured in *B. tabaci* after exposure to a temperature shock? And 3) is any sort of relation between symbiont titres and the Hsp gene expression? Many studies indicate such complex interactions in insects worldwide; however, this study represents an important step in emphasizing possible mechanisms for developing thermal resistance in *B. tabaci*, which is responsible for its sudden outbreak and wide spread in the country, and suggesting new management strategies.

## 2 Materials and methods

### 2.1 Whitefly rearing

Whitefly adults were collected from a research farm (C-Block) in B.C.K.V, India, and reared on brinjal seedlings (Samrat), and the population was maintained in insect-proof rearing cages in the glasshouse under controlled environmental conditions at 26°C ± 1°C with 60% R.H and 16 h light/8 h dark condition and maintained for two generations.

### 2.2 Genetic identification of whitefly and their symbiont

The genetic purity was verified by every generation by molecular analyses. An mt-COI gene was used for the confirmation of whitefly by using forward primer C1-J-2195 (5′-TTG​ATT​TTT​TGG​TCA​TCC​AGA​AGT-3′) and reverse primer L2-N-3014 (5′ TCC​AAT​GCA​CTA​ATC​TGC​CAT​ATT​A-3′) (Simón et al., 1994). After the confirmation of the species, samples were drawn from these pure cultures. Total DNA was extracted using GSure^®^ Insect DNA Mini Kit (GCC Biotech, India) from whitefly samples. The presence of four endosymbionts (*Candidatus Portiera*, *Wolbachia*, *Arsenophonus*, and *Rickettsia*) was detected in the reared whitefly populations using their specific primers (Raina et al., 2015). The presence of these endosymbionts in the field population of whitefly was confirmed with the findings of Singh et al., (2012). A polymerase chain reaction (PCR) program was carried out in a total volume of 25 µl, containing 2 µl of template DNA, 12.5 µl PCR Master Mix, 8.5 µl molecular grade water, and 1 µl each of a forward and reverse primer specific to the symbiont. A thermal cycler programmed a denaturation at 94°C for 5 min, followed by 40 cycles of 94°C for 30 s, annealing at different temperature specific to the endosymbiont (60°C for *Portiera*, 54°C for *Arsenophonus*, and 56°C for *Wolbachia* and *Rickettsia*) for 30 s. An extension was carried out at 72°C for 40 s with a final extension at 72°C for 5 min.

### 2.3 Thermal stress on whitefly

Whitefly adults collected from brinjal plants were placed in small glass tubes of 50 × 5 mm and covered with gauze at the top for smooth breathing. They were subjected to different temperature treatments (T1 = 12°C, T2 = 18°C, T3 = 44°C, and C = 26°C) in a Merck incubator for 3 h, with C being the control for the experiment. Each treatment consisted of three replicates with 20 adult whiteflies in each replicate. The mortality rate of whitefly adults in each replicate was calculated at an interval of 30 min up to 3 h. The tubes were simultaneously transferred at room temperature (26°C) to allow the adult whiteflies to recover the heat shock. The treated samples were henceforth stored at −80°C for further experimentation.

### 2.3 RNA isolation and cDNA synthesis

RNAs were extracted from treated whitefly using the insect RNA isolation kit (Thermo Fisher Scientific) following the manufacturer’s protocol (Morin et al., 2017). For each treatment, the RNA templates consist of 40 individual whiteflies that were eluted in 30 µl of molecular-grade water. RNA quality was evaluated using Invitrogen ™ Qubit ™ four Fluorometer (Thermo Fisher Scientific) to determine the quality and quantity with high precision per µl of RNA, and the eluted templates were stored at −80°C until use.

The synthesis of complementary DNA was performed by using GeneSure H-Minus First-Strand cDNA Synthesis Kit (Genetix Biotech Asia Pvt. Ltd.) by mixing 2.5 μl of total RNA with 1 μl of oligo dT, 1 μl 10 mM dNTPs, and DEPC-treated water to a volume of 12 μl. The solution was incubated at 65°C for 5 min, and the following reagents were added: 4 μl 5X First-Strand buffer, 1 μl ribonuclease inhibitor (40 units/μl), and 4 μl DEPC-treated water. This mixture was placed at 25°C for 5 min before adding 1 μl M-MLV RT. A final incubation at 42°C for 60 min, followed by 70°C for 15 min was performed for terminating the reaction.

### 2.4 DNA extraction

Heat shock-treated whitefly samples (20 individuals/treatments) were subjected to a DNA extraction with the help of the insect DNA extraction kit (GCC Biotech, India). The purified DNA template was eluted in 40 μl of nuclease-free water supplied with the kit. The final products were assessed with the help of Invitrogen ™ Qubit ™ 4 Fluorometer (Thermo Fisher Scientific) to determine the quality and quantity with high precision per µl of DNA.

### 2.5 Quantitative PCR and quantitive RT-PCR analysis

The expression of Hsp genes and the relative amount of different symbionts were examined using the qPCR and qRT-PCR protocol. 2X SYBR Green qPCR Master Mix (Applied Biosystems, United States) was used. Primers name, annealing temperature, and sequences are shown in Table 1. The DNA and cDNA samples were run in triplicate to ensure the validity of the data using the Agilent Technologies Stratagene Mx3000P sequence detection system. Amplification was carried out in 20 µl reaction containing 10 µl 2X SYBR Green PCR Master Mix, 1 µl of each primer (10 µM each), 2 µl template DNA, 0.4 µl ROX, and 5.6 µl molecular-grade water. The cycling condition was as follows: 3 min activation at 95°C, followed by 40 cycles of 40 s at 95°C, 40 s at 60°C, and 45 s at 72°C. The relative expression of each target was calculated using the 2^−ΔΔCT^ method (Livak and Schmittgen, 2001). The *β*-actin (nuclear gene) level, which did not reflect any significant difference across treatments, was used as an endogenous control.

**TABLE 1:** Primers used in the current study. The primer name, accession number, primer sequence, and annealing temperature are listed in the table.

Organism	Accession number	Primer name	Primer sequence (5′→3′)	Annealing temperature (°C)
PCR primers				
“*Candidatus Portiera aleyrodidarum*”	OK036339, OK036338	Por-F	CGT​ACG​GAA​ACG​TAC​GCT​AA	60
		Por-R	TAA​GCA​TAG​GGC​TTT​CAC​ATA​AA	
*Rickettsia* sp.	OK036575, OK044137	Ric-F	GCTCAGAACGAACGCTGG	56
		Ric-R	GAAGGAAAGCATCTCTGC	
*Wolbachia*	OK042301, OK042302	Wol-F	CGG​GGG​AAA​ATT​TAT​TGC​T	56
		Wol-R	AGC​TGT​AAT​ACA​GAA​AGG​AAA	
*Arsenophonus*	OK042289, OK042290	Arse-F	CGT​TTG​ATG​AAT​TCA​TAG​TCA​AA	54
		Arse_R	GGT​CCT​CCA​GTT​AGT​GTT​ACC​CAA​C	
*B. tabaci*	MZ973007, MZ973008	C1-J-2195	TTG​ATT​TTT​TGG​TCA​TCC​AGA​AGT	53
		L2-N-3014	TCC​AAT​GCA​CTA​ATC​TGC​CAT​ATT​A	

**qPCR **	**Target gene**	**Primer name**	**Primer sequence (5′→3′)**	**Annealing temperature (°C)**
“*Candidatus Portiera aleyrodidarum*”	16S rDNA	Port73-F	TAGTCCACGCTGTAAACG	60
		Port266-R	AGGCACCCTTCCATCT	
*Rickettsia sp.*	gltA	glt375-F	AAA​GGT​TGC​TCA​TCA​TGC​GTT	60
		glt574-R	GCC​ATA​GGA​TGC​GAA​GAG​CT	
*Arsenophonus*	23S rDNA	23S-F	CGT​TTG​ATG​AAT​TCA​TAG​TCA​AA	60
		23S-R	GGT​CCT​CCA​GTT​AGT​GTT​ACC​CAA​C	
*Wolbachia*	Wsp	Wsp-F	TGG​TCC​AAT​AAG​TGA​TGA​AGA​AAC	60
		Wsp-R	AAA​AAT​TAA​ACG​CTA​CTC​CA	
*B. tabaci*	Hsp40	Hsp40-F	AGA​TGA​GGC​TCA​TGG​TCA​A	60
		Hsp40-R	TGA​GAA​GCG​CAT​TGC​ATT​GT	
*B. tabaci*	Hsp70	Hsp70-F	ATT​GAA​AAG​TCC​ACT​GGT​AAA​GAA	60
		Hsp70-R	GCT​TGT​ACT​TTT​CAG​CAT​CAG​AC	
*B. tabaci*	Hsp90	Hsp90-F	TGG​AAA​TCA​ACC​CTG​ACC​ACC​CTG	60
		Hsp90-R	TCA​CTG​ACT​TGT​CGT​TCT​TC	
*B. tabaci*	β-actin	Actin-F	ACC​GCA​AGA​TTC​CAT​ACC​C	60
		Actin-R	CGCTGCCTCCACCTCATT	

### 2.6 Data analysis

The differences in the relative expression of Hsp genes and the amount of different symbionts in *B. tabaci* treated under different heat stress conditions were analysed using one-way analysis of variance (ANOVA). The means were compared using Tukey’s test at *p*-value < 0.05. The statistical analysis was performed using SPSS 14.0 (SPSS Inc. Chicago, IL). The error bars present in the graphs represented the standard error.

## 3 Results

### 3.1 Effect of heat stress on adult mortality

**FIGURE 1:**
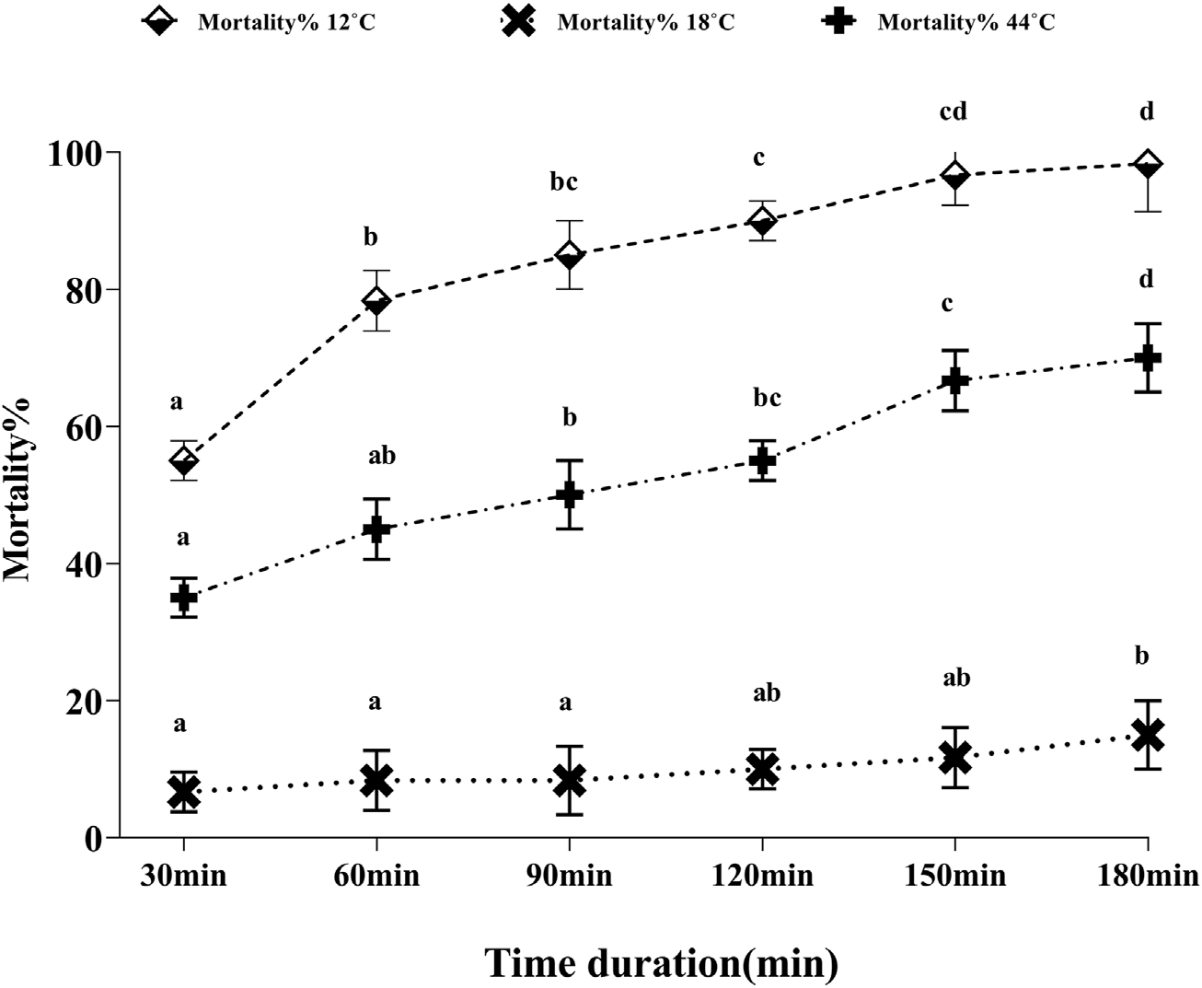
Percentage mortality of whitefly adults in response to thermal stress (12°C, 18°C, and 44°C). Whitefly adults from each treatment were incubated for 30, 60, 90, 120, 150, and 180 min in three replicates, and the mortality rates were measured after each incubation time.

To evaluate the direct effect of temperature stress in whiteflies, the mortality rate of the whitefly adults was counted every 30 min of exposure for 3 h (Figure 1). Upon the exposure of whitefly to 44°C, the initial mortality rate was noted to be 35%, which steadily increased to 66.67% and 70% at 2.5 and 3 h, respectively. On the contrary, at an extremely low temperature (12°C), the mortality rate was calculated to be as high as 55% within the initial 30 min that rapidly increased to 78% after 1 h of continuous heating, and finally reached 98.33% until 3 h. However, at a moderately low temperature (18°C), the calculated mortality rate was low, 6.67% in the initial 30 min to only 15% until 3 h.

### 3.2 Characterization of cryptic species of *B*. *tabaci* and the endosymbionts

**FIGURE 2:**
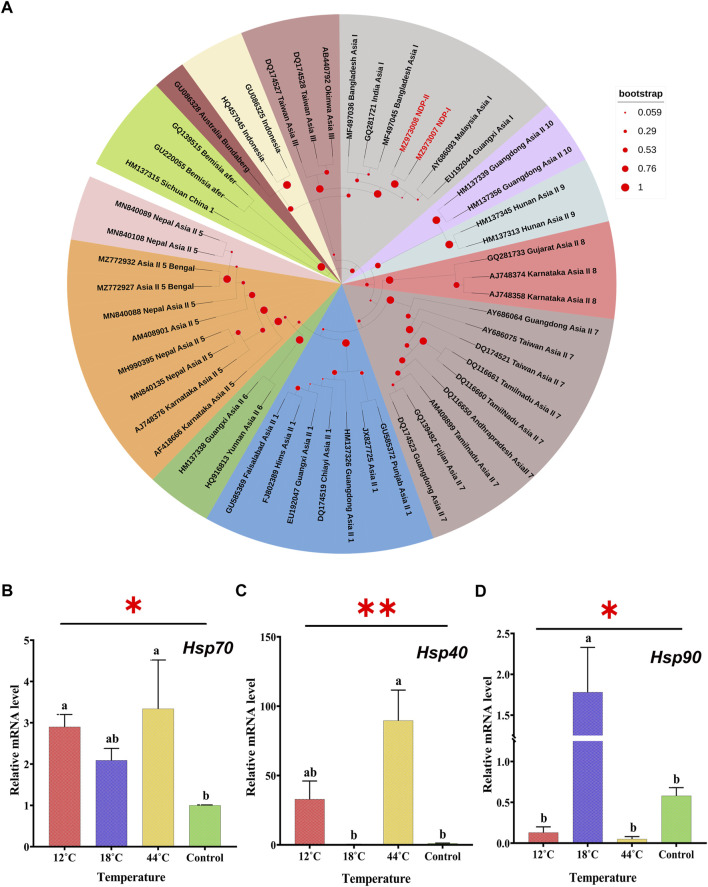
**(A)** Phylogenetic tree of *B*. *tabaci* cryptic species is identified based on cytochrome oxidase subunit I (COI) sequences. The samples from the study are indicated by bold text (red) in the tree; all other sequences were obtained from the GenBank database. *Bemisia afer* sequences were taken as an out-group. Effect of thermal stress (12°C, 18°C, 26°C, and 44°C) on the transcript level of Hsp70 **(B)**, Hsp40 **(C)**, and Hsp90 **(D)**. Adult whitefly exposed to 26°C was considered as control. Relative mRNA expression levels measured by qRT-PCR with β- actin are used as a reference gene. The different letters indicate statistically significant differences between the treatments. *p* ≤ 0.05 is indicated by *, and *p* ≤ 0.01 is indicated by **.

Several DNA-based techniques have been exploited for proper identification of *B*. *tabaci* cryptic species (Simón et al., 1994). Nonetheless, sequence analysis of the mitochondrial cytochrome oxidase I (mt-COI) gene has been the most widely accepted (Barman et al., 2022c). In the current study, running culture is one homogenous population of *B*. *tabaci* that was identified by using the primer pair (C1-J-2195 F/L2-N-3014 R) of the universal mt-COI gene. Based on the previously known sequences in the GenBank database, a phylogenetic tree was constructed by using the maximum likelihood phylogram (Figure 2A). The phylogenetic analysis of the determined COI sequences assured that the populations belonged to Asia-I cryptic species. The sequence can be retrieved using the GenBank Accession No. MZ973007 and MZ973008.

**FIGURE 3:**
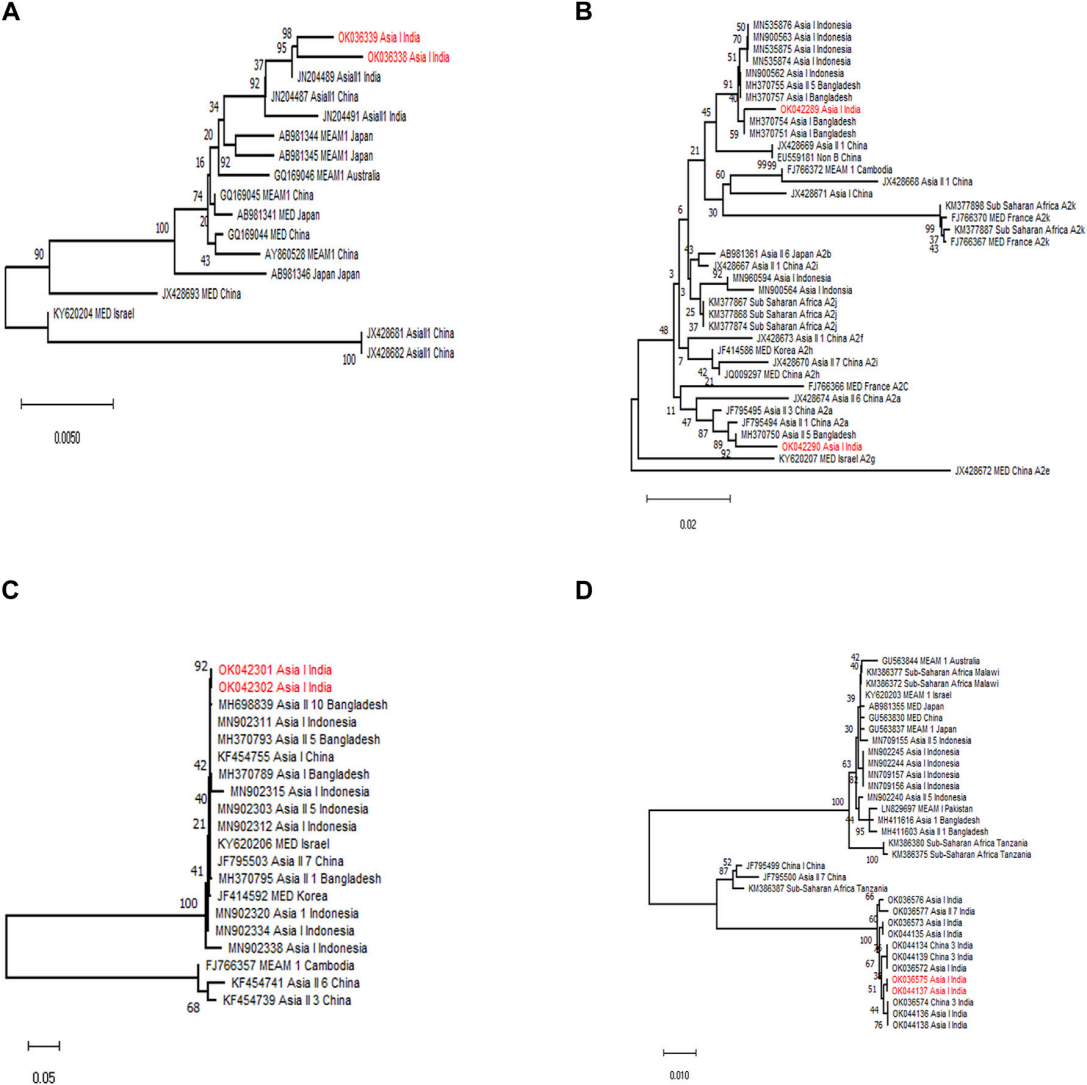
Phylogenetic tree of primary and secondary endosymbionts of *B*. *tabaci* based on 16S rDNA (*Portiera*, *Wolbachia*, and *Rickettsia*) and 23S rDNA (*Arsenophonus*) gene segments. The samples from the study are indicated by bold text (red) in the tree; all other sequences were obtained from the GenBank database. **(A)** Maximum likelihood phylogenetic tree of the 16S rDNA sequences of *Portiera* sp. infecting different whitefly populations. **(B)** Maximum likelihood phylogenetic tree of the 23S rDNA sequences of *Arsenophonus* sp. infecting different whitefly populations. **(C)** Maximum likelihood phylogenetic tree of the 16S rDNA sequences of *Wolbachia* sp. infecting different whitefly populations. **(D)** Maximum likelihood phylogenetic tree of the 16S rDNA sequences of *Rickettsia* sp. infecting different whitefly populations.

Subsequently, a diagnostic PCR confirmed the presence of primary endosymbiont *Portiera* and secondary endosymbionts *Wolbachia*, *Arsenophonus*, and *Rickettsia* in the selected whitefly population. The sequencing results of the products could generate 1,350, 580, 560, and 800 nt sequences for *Portiera*, *Arsenophonus*, *Wolbachia*, and *Rickettsia*, respectively. From BLASTn analysis, we obtained 100% similarity with other sequences available in NCBI (Figure 3).

### 3.3 Effect of temperature treatments on an Hsp transcript level

After the exposure of whitefly at 12°C, 18°C, and 44°C for 3 h (hours), the transcript level of Hsp40, Hsp70, and Hsp90 displayed mercurial changes in their expression pattern. The transcript level of Hsp70 showed significant (F_3, 8_ = 2.687, *p* = 0.017) upregulation under all the three temperature conditions with an increase of 2.90-, 2.09-, and 3.34-fold (Figure 2B), whereas the transcript level of Hsp40 was not upregulated at all three temperature regimes but only at 12°C and 44°C with an increase of 32.85- and 89.62-fold, respectively. Moreover, Hsp40 showed noticeable downregulation of the transcript level at 18°C (F_3,8_ = 10.71, *p* = 0.003) (Figure 2C). On the contrary, the expression level of Hsp90 was downregulated at extremely low (12°C) and high (44°C) temperatures with an elevation of 1.78 times, observed at 18°C which showed statistical significance (F_3,8_ = 7.83, *p* = 0.009) (Figure 2D).

### 3.4 Relative density of endosymbionts in whitefly after different temperature treatments

**FIGURE 4:**
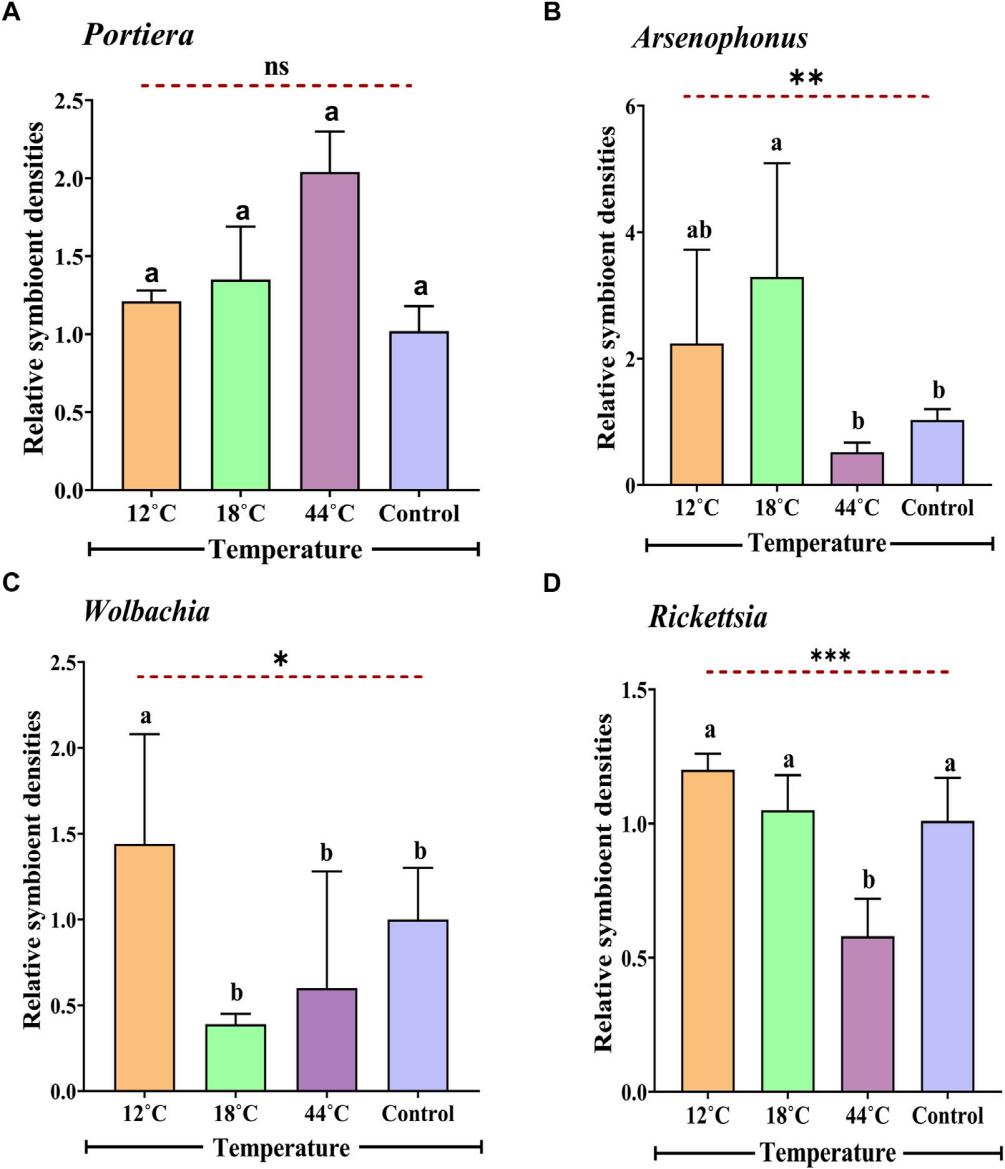
Relative titre of *Portiera* **(A)**, *Arsenophonus* **(B)**, *Wolbachia* **(C)**, and *Rickettsia* **(D)** in four different temperatures (12°C, 18°C, 26°C, and 44°C) *B*. *tabaci* populations as determined by quantitative PCR (normalized according to the amount of an actin gene). Adult whitefly exposed to 26°C was considered as control. Values for the relative amount of symbionts are means ± SEM of three replicates for each treatment. The data were analysed with one-way ANOVA. The different letters indicate statistically significant differences between the treatments. *ns* = non-significant, *p* ≤ 0.05 is indicated by *, *p* ≤ 0.01 is indicated by **, and *p* ≤ 0.001 is indicated by ***.

After exposing whitefly to different temperature treatments (12°C, 18°C, and 44°C), the relative titre of four endosymbionts (*Candidatus Portiera*, *Arsenophonus*, *Wolbachia*, and *Rickettsia*) was measured. It was observed that the primary and secondary symbiont titre were markedly different in terms of relative quantity (Figures 4A–D). In the presence of extremely high temperatures (44°C), the primary endosymbiont (*Portiera*) was 2.04-fold greater in the relative amount, whereas the three secondary endosymbionts, namely, *Arsenophonus*, *Wolbachia*, and *Rickettsia*, had a reduction in the relative density (0.62-, 0.68-, and 0.58-folds, respectively) as compared to the control. Significant differences were observed for all the secondary symbionts (*Arsenophonus*: F _3, 8_ = 13.39, *p* = 0.0017; *Wolbachia*: F _3, 8_ = 4.34, *p* = 0.041; and *Rickettsia*: F_3,8_ = 19.27, *p* = 0.0005) except the primary symbiont *Portiera* (F _3, 8_ = 1.07, *p* = 0.41). There was an increase in relative densities of 1.35-fold in *Portiera*, 3.29-fold in *Arsenophonus*, and 1.05-fold in *Rickettsia* at 18°C, whereas the relative densities of *Wolbachia* exhibited a decrease of 0.39-fold. Alternatively, at extremely low temperatures (12°C), all aforementioned endosymbionts showed an increase in the relative density.

### 3.5 Correlation of a relative endosymbionts titre with Hsp gene expression at different temperature treatments

**FIGURE 5:**
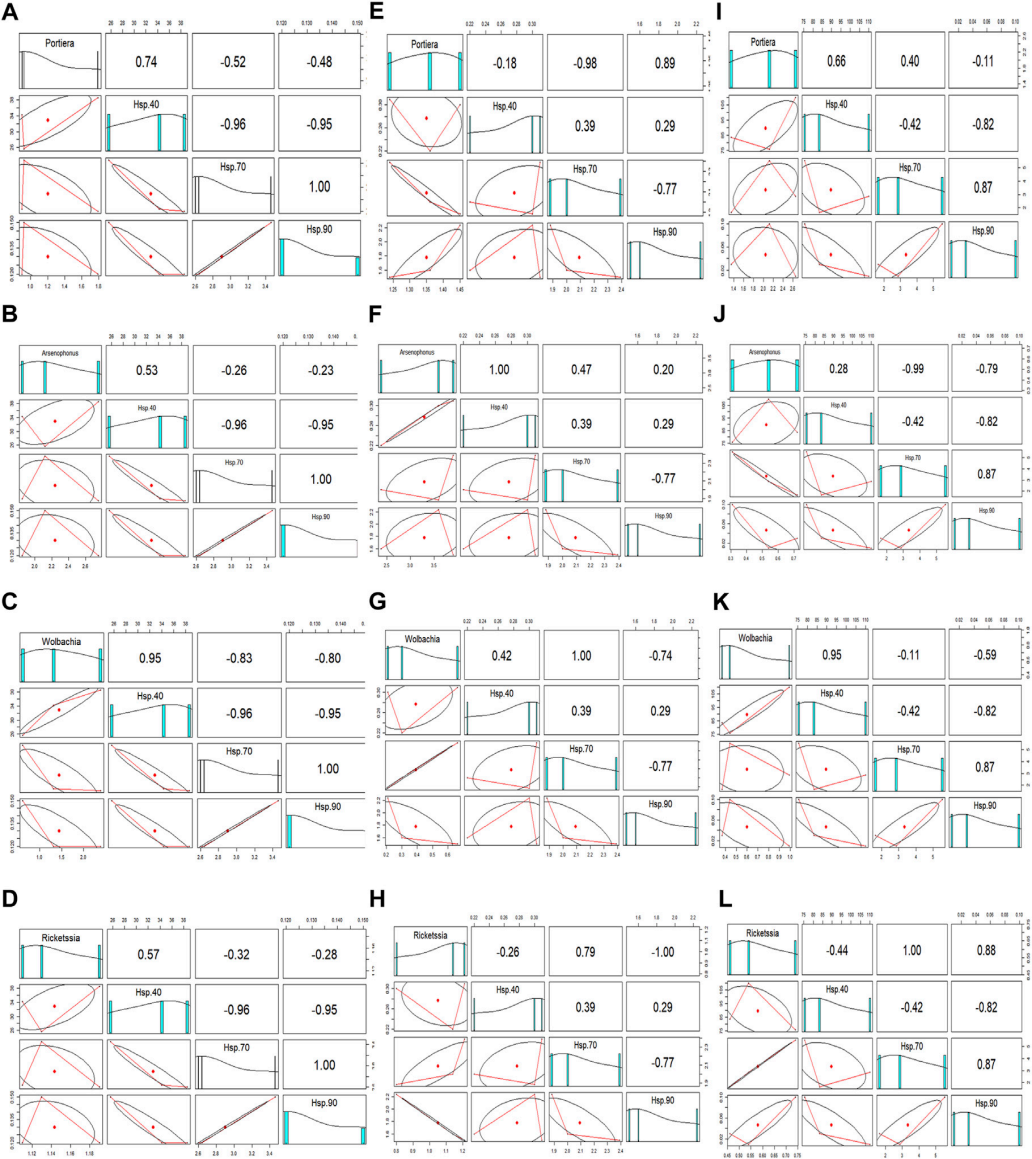
Correlation of relative endosymbiont (*Portiera, Arsenophonus, Wolbachia*, and *Rickettsia*) titres and Hsp gene (Hsp40, Hsp70, and Hsp90) expression, at **(A–D)** 12°C, **(E–H)** 18°C, and **(I–L)** 44°C.

Hsp gene expression correlated positively or negatively with the relative endosymbiont titre at different temperatures (Table 2). There was a strong uphill linear relationship between all the endosymbionts and Hsp40 at extremely low temperatures (12°C) (Figures 5A–D), whereas both Hsp70 and Hsp90 expression showed a downward linear relationship with all four endosymbionts. The *Wolbachia* titre is highly influenced at extremely low temperatures (12°C) by Hsp expression, while the *Arsenophonus* titre is least affected, either positively or negatively. At moderately low temperature (18°C), *Arsenophonus* and *Wolbachia* displayed a positive relationship with Hsp40, whilst *Portiera* exhibited a negative relation. In a similar manner, all the secondary symbionts (*Arsenophonus*, *Wolbachia*, and *Rickettsia*) except *Portiera* exhibited a positive relation with Hsp70. Nonetheless, primary symbiont, *Portiera*, exhibited a strong uphill linear relationship with Hsp90. Thus, at a moderately low temperature (18°C), the relationship between symbiont titres and Hsp gene expression varied significantly (Figures 5E–H). Lastly, at an extremely high temperature (44°C), the relative titre of *Portiera*, *Arsenophonus*, and *Wolbachia* showed a positive relationship with Hsp40, whereas *Rickettsia* exhibited a negative relation. On the contrary, the relative titre of *Rickettsia* exhibited a positive correlation with Hsp70 and Hsp90, while *Arsenophonus* and *Wolbachia* displayed an opposite trend (Figure 5I–L).

**TABLE 2:** Correlation of relative endosymbiont titres and Hsp gene expression, under different temperature treatments.

Temperature	r	P	*R* ^2^	r	P	*R* ^2^	r	P	*R* ^2^
Treatment	Hsp40			Hsp70			Hsp90		
Relationship with *Portiera* titre									
12°C	0.74	0.46	0.54	−0.52	0.65	0.26	−0.48	0.67	0.23
18°C	−0.18	0.88	0.03	−0.98	0.03*	0.95	0.89	0.04*	0.78
44°C	0.66	0.54	0.43	0.40	0.73	0.16	−0.11	0.93	0.01
Relationship with *Arsenophonus* titre									

Temperature	r	P	R^2^	r	P	R^2^	r	P	R^2^
Treatments	Hsp40			Hsp70			Hsp90		
12°C	0.53	0.64	0.28	−0.26	0.82	0.07	−0.23	0.85	0.05
18°C	1.00	0.04*	0.99	0.47	0.68	0.22	0.20	0.86	0.04
44°C	0.28	0.81	0.08	−0.99	0.09	0.97	−0.78	0.42	0.61
Relationship with *Wolbachia* titre									

Temperature	r	P	R^2^	r	P	R^2^	r	P	R^2^
Treatments	Hsp40			Hsp70			Hsp90		
12°C	0.95	0.19	0.90	−0.83	0.38	0.68	−0.80	0.40	0.64
18°C	0.42	0.72	0.17	0.99	0.02*	0.99	−0.74	0.46	0.55
44°C	0.95	0.02*	0.90	−0.11	0.92	0.012	−0.59	0.59	0.35
Relationship with *Rickettsia* titre									

Temperature	r	P	R^2^	r	P	R^2^	r	P	R^2^
Treatments	Hsp40			Hsp70			Hsp90		
12°C	0.57	0.61	0.32	−0.32	0.79	0.10	−0.28	0.82	0.07
18°C	−0.26	0.83	0.06	0.79	0.42	0.62	−1.00	0.02*	0.99
44°C	−0.44	0.70	0.19	1.00	0.01*	0.99	0.88	0.31	0.77

r, correlation coefficient; R^2^, coefficient of determination; and *, significant level (*p* < 0.05).

## 4 Discussion

Temperature is one of the important determinants of an organism’s distribution and abundance (Colinet et al., 2010). The ability to tolerate thermal stress is a vital parameter enabling the survival of *B. tabaci* under varying temperature conditions, thus playing a pivotal role in its wide distribution pattern in the Indian sub-continent (Samanta et al., 2021). In the current study, we determined the mortality rate of whitefly adults on the exposure to thermal stress and highlighted the molecular aspects underlying the heat responsive mechanism in *B. tabaci.* The varying mortality percentage of whitefly on subjection to different temperature stress conditions was observed with a higher mortality rate at 12°C than the mortality rate noted at 44°C. Cui et al. (2008) also reported a low mortality rate of whitefly at higher temperatures (40°C). A possible reason for this variation may be the differential expression pattern of Hsp genes that are reported to play a vital role in protecting organisms under heat stress conditions (Salvucci et al., 2000; Hoffmann et al., 2003; Wang et al., 2019).

Hsp genes are a central protagonist in helping organisms to cope with different environmental challenges, such as pathogen infection, xenobiotic substances, and thermal stress conditions (Chakraborty et al., 2021; Derecka et al., 2013; Somero, 1995; Sun and MacRae 2005; Tissiêrs et al., 1974). The present study puts forward the differential expression of three Hsp genes (Hsp40, Hsp70, and Hsp90) in *B. tabaci* under a heat-shock condition. The temperature treatment of whitefly resulted in an induced expression of Hsp70 under all the three conditions (12°C, 18°C, 44°C, and 26°C acting as control) with maximum expression observed at 44°C. The elevation of Hsp70 at extremes of temperature suggests its involvement in both heat and cold adaptation (Xiao et al., 2019). Reports suggest that the optimal expression level of Hsp70 is critical to the maintenance of cell function and homeostasis (Wheeler et al., 1999; Kristensen et al., 2002) and for chaperons to bind peptide chains (Fink, 1999). The transcript level of Hsp40 was also significantly upregulated upon the exposure of whitefly to extreme cold (12°C) and hot (44°C) conditions with a much higher expression level at 44°C, indicating the possible involvement of Hsp40 in heat adaptation of whitefly. Previous studies mentioned the association of Hsp genes in insects with important functions such as the regulation of growth and reproduction that, henceforth, increased their ability to fit under adverse environmental conditions (Lu et al., 2015; Quan et al., 2022). Work pertaining to the role of Hsp40 in whitefly seems limited; however, there are unequivocal indications regarding the role of Hsp40 in Hymenoptera, wherein, upon protein denaturation, Hsp40 delivers unfolded protein to Hsp70, and they together facilitate refolding by ATP binding and hydrolysis (Nguyen et al., 2016; Rinehart et al., 2007). Upregulation of the two Hsps (Hsp40 and Hsp70) under thermal stress conditions are indicative of their role in preventing cell damage under such stress conditions.

Contrary to the upregulation of most Hsp genes subjected to heat stress, a low basal expression of Hsp90 was observed, when exposed to extremes of temperature. Hence, it would not be wrong to say that the expression of Hsp90 is less dependent upon temperature stress than other Hsps. However, in the case of Hsp90, the transcript level attained a peak at 18°C. This clearly suggests that Hsps might have evolved from different expression patterns under different temperature conditions. Reports indicate the participation of Hsp90 in the negative regulation of proteins (Lindquist and Craig, 1988). The susceptibility of *B. tabaci* to temperature has been reported to vary according to geography and genetic groups (Pusag et al., 2012). This highlights the importance of thermal tolerance for insects including whitefly to thrive under such a diverse climatic condition as that of India.

In addition to determining the role of Hsp genes in adaptation and survivability of whitefly, an important dynamic which remains unattended is the host–microbe association or to say symbiont-mediated modulation of host traits such as thermal tolerance. Thermal variation has a strong influence on host metabolism, and any deviation from optimum environmental conditions could have a deleterious influence on the hosts’ survival and fecundity (Macmillan, 2019). To shed light on this aspect, we evaluated the change in the relative amount of four endosymbionts in whitefly when subjected to thermal stress. Although we are aware that many host–microbe interactions protect their host partners from pathogens or predators (Flórez et al., 2015), less is acknowledged regarding the influence of these symbionts on insects’ thermal tolerance. However, reports suggest that temperature has a significant influence in the abundance of endosymbionts harboured in insects and their interaction (Bensadia et al., 2006; Burke et al., 2010.; Dunbar et al., 2007.; Montllor et al., 2002). Microbes can either expand or restrict the abiotic niche of their host partners, thus influencing their ability to adapt to the fluctuating environmental condition (Lemoine et al., 2020; Zaynab et al., 2019).

Our findings revealed multiple patterns in the relative abundance of primary and secondary symbionts upon subjection to thermal stress. The primary endosymbiont, *Portiera*, was relatively unaffected by temperature treatment. Localization of *Portiera* inside the bacteriosome might be held accountable for their limited response to temperature stress (Su et al., 2014; Li et al., 2017; Caspi-Fluger et al., 2011). On the contrary, depletion in the quantity of the three secondary symbionts (*Arsenophonus*, *Wolbachia*, and *Rickettsia*) was measured at 44°C. This indicates a possible involvement of the facultative symbionts in enhanced thermo-tolerance of *B. tabaci* at extremely high temperatures and hence the low mortality rates at this temperature. Experiments carried out in the pea aphid also demonstrated the involvement of facultative symbionts in protecting hosts from detrimental effects of thermal stress (Oliver et al., 2010). Brumin et al. (2011) reported the spatial location of *Rickettsia* outside the bacteriosome, resulting in a significant decrease of the endosymbiont when subjected to thermal stress. Shan et al. (2014) also observed a reduction in *Rickettsia* population in heat-treated whitefly, although the reduction was non-significant. Disturbances in gut symbionts when exposed to high temperature were noted in many insects. A possible explanation to this could be that an escalation in temperature upsets the stability of some protein that is involved in the transportation of metabolite, thereby restricting supplementation of the metabolites between the partners. It was also seen that titres of *Wolbachia* were depleted in *Aedes albopictus* when exposed to elevated temperature conditions (Mouton et al., 2007; Wiwatanaratanabutr and Kittayapong, 2009). Such depletion does not essentially indicate host extinction. Hence, the primary symbionts are reported to be directly involved in vital metabolic functions of insect host, and on the flip side, the secondary symbionts are reported to mitigate the effect of heat stress on the primary symbionts, thus aiding the hosts’ survival (Lemoine et al., 2020). Thus, it would not be wrong to say that host–microbial association has a key role in temperature acclimation; however, the exact dynamics vis-à-vis direct effect of an abiotic factor on any sort of symbiotic interactions still remains unknown and will require further exploration.

Deleterious effects of thermal stress on symbiont quantity may be allayed *via* other mechanism such as gene expression (Brumin et al., 2011) or modulation of the host behaviour (Truitt et al., 2019). In the current study, the expression of the Hsp genes and relative symbionts titre were highly variable under different temperature conditions. Based on the correlation studies, we assumed that endosymbionts infection modulate host gene expression by altering the expression of specialized Hsp genes or by affecting the activities of transcription factors (Moon et al., 2021; Brennan et al., 2008). In addition, further investigation is required in this direction.

In the light of current disturbances in the ecological and environmental balance caused by human activities, the present investigation can be considered an important step in predicting the potential factors responsible for the adaptation of whitefly in such a diverse temperature regime. However, few vital questions that arise on our way stem from the basic evolution of symbiont-mediated thermal resistance/heat tolerance and the precise molecular interactions occurring between symbionts and host genes. Particularly, through proper clarification of these vital issues, a symbiont-targeted pest management will prove to be an effective control component for the future agricultural community. However, detailed work needs to be carried out before the large-scale application of symbiont-targeted pest management strategies.

## Data Availability

The datasets presented in this study can be found in online repositories. The names of the repository/repositories and accession number(s) can be found in the article/Supplementary Material.
